# Inter-subject variability modulates phonological advance planning in the production of adjective-noun phrases

**DOI:** 10.3389/fpsyg.2014.00043

**Published:** 2014-01-31

**Authors:** Violaine Michel Lange, Marina Laganaro

**Affiliations:** Faculty of Psychology and Educational Sciences, University of GenevaGeneva, Switzerland

**Keywords:** speech production, phonological encoding, speech processing, advance planning, variability

## Abstract

The literature on advance phonological planning in adjective-noun phrases (NPs) presents diverging results: while many experimental studies suggest that the entire NP is encoded before articulation, other results favor a span of encoding limited to the first word. Although cross-linguistic differences in the structure of adjective-NPs may account for some of these contrasting results, divergences have been reported even among similar languages and syntactic structures. Here we examined whether inter-individual differences account for variability in the span of phonological planning in the production of French NPs, where previous results indicated encoding limited to the first word. The span of phonological encoding is tested with the picture-word interference (PWI) paradigm using phonological distractors related to the noun or to the adjective of the NPs. In Experiment 1, phonological priming effects were limited to the first word in adjective NPs whichever the position of the adjective (pre-nominal or post-nominal). Crucially, phonological priming effects on the second word interacted with speakers' production speed suggesting different encoding strategies for participants. In Experiment 2, we tested this hypothesis further with a larger group of participants. Results clearly showed that slow and fast initializing participants presented different phonological priming patterns on the last element of adjective-NPs: while the first word was primed by a distractor for all speakers, only the slow speaker group presented a priming effect on the second element of the NP. These results show that the span of phonological encoding is modulated by inter-individual strategies: in experimental paradigms some speakers plan word by word whereas others encode beyond the initial word. We suggest that the diverging results reported in the literature on advance phonological planning may partly be reconciled in light of the present results.

## Introduction

Language production is a complex and generative process during which cognitive processes unfold in time from concept to articulation (Levelt, 1989). Although words represent the building blocks of sentences, it is generally considered that speakers probably do not encode one word after the other since this would result in scattered, disfluent speech. Nevertheless, it is unlikely that speakers have planned an entire sentence before they start articulating the first lexical word, since this would involve long speech breaks and memory overload. Furthermore, the amount of advance planning is probably not fixed and it may vary according to various linguistic (language, syntax, etc.) and extra-linguistic (speaker's age, speed, stress, etc.) factors. Here we aim at investigating to what extent the span of phonological encoding varies across speakers for adjective-NPs.

To do so, we first describe how speech production models deal with the question of the amount of advance planning. Then we review how non-experimental data can provide information on this question. Finally, we focus on experimental paradigms and on the contradictory results emerging from the literature.

Most language production models usually agree on the main processing stages involved in word production (Dell, 1986; Levelt et al., 1999), although they do not always agree on the flow of activation across the system, nor on the way speakers plan ahead before speaking. After the activation of a pre-linguistic concept, the formulation process involves semantic and grammatical encoding, where the different thematic roles are attributed a functional role (e.g., subject, object). The second stage in the formulation processes, lexical processing, involves the selection of the lexical entries (lemmas) corresponding to the concepts (lexical-semantic encoding) and the retrieval/encoding of the lexemes, i.e., of the corresponding phonological codes. Finally, the articulatory plans can be prepared and articulation initiated. The question of how much speakers plan ahead at these different encoding stages is essential to understanding language production mechanisms. The amount of advance planning has been addressed in particular in serial models of language planning (Levelt, 1989), where it has been proposed to be larger at the grammatical and lexical levels than at the level of phonological encoding. No matter how much has been encoded at previous encoding levels, the speech system will only process one phonological word at a time during phonological encoding. The phonological word, which represents the unit of encoding at the phonological level according to Levelt (1989), is often defined as a stressed word and all the unstressed words that attach to it. In Levelt's view, the encoding unit at the phonological level is and remains fixed no matter the content of the message or discourse constraints.

However, this proposal has been challenged by some results reported in the literature. The experimental data on the span of encoding in the production of multiword sentences are extremely divergent, including results favoring a minimal amount of ahead planning (e.g., Meyer, 1996) and claims that an entire multiword sentence can be planned before articulation (e.g., Schnur et al., 2006; Oppermann et al., 2010; Schnur, 2011). Several reasons for these diverging results have also been sketched. First, the amount of ahead planning may differ across languages, as these diverging experimental results involved very different languages (e.g., Romance vs. Germanic languages). Second, very different experimental paradigms are used to investigate the same question, which might create artifacts that researchers are still unable to control. This issue has been underlined in several recent reports (Oppermann et al., 2010; Jaeger et al., 2012; Damian et al., under revision). An additional clue is that the amount of advance planning may vary across speakers and this variability may be missed in an experimental context. As a result, speakers' variability is seldom taken into account in studies investigating advance planning even though it has been reported to affect the speech encoding processes (Wagner et al., 2010; Gillespie and Pearlmutter, 2011). In sum, different factors could affect the span of encoding in the production of multiword sentences. In the following we will focus on whether cross-linguistic differences and/or inter-individual differences best account for phonological encoding variability.

## Speech errors and sandhi phenomena as indicators of advance planning

The earliest source of information concerning the extent of advance planning in language production was the study of speech errors (see Fromkin, 1973; Garrett, 1975, 1980; Meyer, 1992). In particular, metathesis and anticipation errors give information on the minimal extent to which a speaker has planned ahead, as the fact that an upcoming word or phoneme is produced at an earlier position in the utterance indicates advance planning at least up to this element. The analysis of speech errors suggested that lexical errors (word exchange errors for instance) can occur in a fairly large span while phonological exchange and metatheses involve segmental units within a much smaller span, often limited to three syllables (Rossi and Peter-Defare, 1998). These observations suggest that the span of grammatical and lexical-semantic encoding may be larger than the span of phonological planning. Recently, in a study by Gillespie and Pearlmutter (2011), the authors analyzed syntactic agreement errors to investigate advance planning in grammatical encoding in sentence production. They made the hypothesis that individuals' difference in speed of speech production and advance planning might influence their sensitivity to agreement errors. They investigated this hypothesis by measuring speech onset latencies and error agreement in a picture description task involving complex NPs. Results showed that speakers who were slower to initiate speech produced more agreement errors, suggesting that slower speakers do more advance planning and are more likely to experience interference during agreement computation probably due to an overload of the encoding system.

Specific syntactic and phonological phenomena such as external sandhi also provide some information on the amount of advance planning in sentence production. This linguistic phenomenon refers to phonological changes occurring at word boundaries in connected speech. For instance, the obligatory *liaison* in French involves the pronunciation of a latent consonant only in specific word boundary conditions (e.g., grand—*great* and ami—*friend* would be pronounced/grã and ami/in isolation but/grã tami/in the NP “great friend” because of the *liaison* phenomenon). This linguistic phenomenon is often found in Romance languages but not in Germanic languages (Nespor and Vogel, 2007) and is obligatory only in a specific context. For instance, French liaisons are obligatory for pre-nominal adjective NPs but not for post-nominal adjective NPs (Stark and Pomino, 2009). Whether a *liaison* is realized or not can be motivated by several components. For instance, syntactic components of the message (Laks, 2005), syntactic cohesion (Bybee, 2001) which is a matter of frequency of co-occurrence and speech context (Encrevé, 1988) condition the realization of a *liaison*. Resyllabification involved in *liaison* sequences represents a major argument for models of speech production which claim that the minimal unit of encoding is not the lexical word but rather the phonological word (Levelt, 1989). The correct pronunciation of a *liaison* sequence requires therefore the phonological encoding of the onset of the following word and suggests that encoding at the phonological level extends the initial lexical word. Thus, when producing French A+N NPs in particular, one may assume that the entire sequence is planned at least up to phonological encoding processes.

## Experimental paradigms to investigate the span of encoding

Different experimental paradigms have been used to test the span of encoding in language production. Alario et al. (2002) and Schnur (2011) for example used lexical frequency effects in picture naming tasks to test the amount of advance planning, with the hypothesis that any effect of lexical frequency reported for a given word suggests that phonological encoding extends to this word. However, as Alario et al. (2002) underline in their study, the locus of the frequency effect in picture naming is still debated and might not reflect what happens at the phonological level but at other encoding levels.

To avoid problems linked to the locus of an effect of a psycholinguistic variable, other authors used priming paradigms. The idea behind these paradigms is that if the latency of production of the first word in a sentence is affected by a prime related to a word coming up later, then one can conclude that encoding extends at least up to the word related to the prime. For example, Meyer (1996), tested word pairs such as *the arrow and the bag* with semantic and phonological distractors for each word of the pair. She obtained an interference effect from the semantic distractors compared to the neutral condition for both elements of the word pairs. By contrast, the facilitation effect from the phonological distractors was observed for the first word of the pair only. She concluded that the span of encoding is wider at the lexical level than at the phonological level.

## The role of syntactic structures in advance planning

Meyer's results provide information about the span of encoding for two simple noun-phrases. However, one can wonder whether encoding of a single but syntactically more complex NP, namely adjective-NPs, gives rise to different encoding patterns. In a cross-linguistic study, Schriefers and Teruel (1999a) investigated advance planning of adjective-NPs at the lexical-semantic level with a priming paradigm. The authors compared the production of NPs in German and in French with semantic distractors. In German, where the adjective is pre-nominal (A+N), the first smallest full syntactic phrase is the entire NP. In French, where the adjective is post-nominal (N+A), the first smallest full syntactic phrase is the determiner + noun. What defines the first smallest full syntactic phrase in this view is the head of the NP (i.e., the noun). In their study, Schriefers and Teruel (1999a) observed an interference effect for both elements in German (A and N in A+N) and a priming effect limited to the noun in French (N in N+A). The authors concluded that these results were in favor of evidence for cross-linguistic variation of grammatical advance planning. What is most relevant for the present study is that the minimal amount of encoding at the lexical-semantic level in French seems to be the first smallest full syntactic phrase. If this is the case, processing of the next grammatical element (here the adjective) should initiate only once the first word (the noun) has been fully encoded. Contrarily, in the case of Germanic languages, encoding processes in NPs seem to be determined by the second element (i.e., the head noun). Deductively, if the span of encoding at the lexical-semantic stage corresponds to the smallest full phrase, one can expect it to be either equivalent or shorter at the phonological processing stage, i.e., equivalent or shorter than the two constituents in A+N, and limited to the first element in N+A.

This hypothesis was tested by Dumay et al. (2009) and later by Damian et al. (under revision) in a cross-linguistic study using the initial phoneme repetition priming paradigm (i.e., phonological priming by repeated onsets such as in *b*lue *b*ag) on different types of NPs. The authors tested one Germanic language (English), where the color adjectives of the NPs are pre-nominal, and two Romance languages (Spanish and French), where the adjectives are post-nominal. As predicted by Schriefers and Teruel (1999a), they observed phonological facilitation of repeated phonemes for English A+N NPs where the head noun was the second element and failed to obtain an effect of phonological facilitation for the Spanish and French experiments where the head noun was the first element. Nevertheless, the authors suggested that their results might be due to the fact that color identification might be more difficult than object identification, therefore affecting differently the results when the color adjective is in first or second position. In a subsequent experiment, they rendered colors more salient and tested participants in English and Spanish. The facilitatory effect of repeated initial phonemes was replicated in English, where the overall naming latencies were shorter relative to the first experiment, where only colored line drawings were used. However, longer RTs were reported for the initial phoneme repetition condition in Spanish. Overall, these results led the authors to argue for a sequential model of encoding with a level of activation slightly higher for the nouns relative to the adjectives. This model explains why a facilitation effect is observed in the English NP (A+N) condition where the adjective will receive extra facilitation from phonological priming with the noun. However, in the Spanish NP condition (N+A), interference will occur from the priming effect of the adjective with the noun in initial position. The authors conclude that their results are not in line with Schriefers and Teruel's (1999a) since they did not observe cross-linguistic differences in the encoding processes but rather similar underlying mechanism of coding for sequential order influenced by a stronger activation of the noun.

Similarly, Costa and Caramazza (2002) ran a cross-linguistic study in English and Spanish testing adjective-NPs in a picture naming task with phonological distractors. In this study, the target word was the last word in the phrase (the noun in English and the adjective in Spanish). Since they obtained a facilitation effect for the prime independently of the language, they concluded that the entire sequence had been encoded at the phonological level before articulation. If all the studies reviewed so far report a priming effect for the N in A+N NPs, at least one study challenges this otherwise reliable effect. Schriefers and Teruel (1999b) tested A+N NPs in German using a phonological priming paradigm. The distractor words primed either the first or second syllable of the first word or the first syllable of the second word. They failed to obtain a facilitation effect on the first syllable of the second word across four experiments. Moreover, they also failed to obtain a facilitation effect for the second syllable of the first word. The authors concluded that the minimal unit of encoding could be smaller than the phonological word.

Although most studies investigated adjective-NPs, which are also our focus here, we will briefly review a few studies investigating the span of phonological encoding beyond NPs. These studies are of particular interest because they seem to indicate that the span of phonological encoding may extend beyond noun-phrases. Schnur et al. (2006) reported phonological priming when the verb was the last element of a sentence such as *The orange girl jumps*. In a subsequent study (Schnur, 2011), similar results were obtained when the last element of the sentence was a noun (e.g., *The girl kicks the ball*). As both a facilitation and a frequency effect of the noun were observed, the author concluded that phonological planning extends across the entire phonological phrase, to both the verb and the following direct object NP. Oppermann et al. (2010) obtained similar results in a study where German participants were shown pictures corresponding to sentences with different syntactic structures and were then asked to remember them and repeat them on the presentation of a cue. Phonological distractors were used at different stimulus onset asynchrony. Phonological priming was reported for the noun in final position in some of the utterance formats tested but not in all of them. The authors concluded that the span of phonological encoding could therefore extend to a single syntactic phrase and maybe to an entire sentence. Contrary to the results reported by Schnur et al. (2006) and Schnur (2011), the effect of the phonological prime in the Oppermann et al. (2010) study was facilitating on the first word while interfering on the last word. Finally, Wagner et al. (2010) investigated whether variability in speakers' speech onset latencies may affect the span of advance planning. Participants were asked to name pictures corresponding to sentences such as *The frog is next to the mug* in a semantic priming paradigm. The results were analyzed according to the participants' production latencies (speakers with “slow” or “fast” latencies). The interference effect of the semantic distractors was much smaller for nouns in the second position for the “fast” group than for the “slow” group. Similarly to Gillespie and Pearlmutter (2011), the authors concluded that fast speakers show a tendency toward incremental grammatical advance planning while slow speakers present full grammatical advance planning of the entire utterance. Except for these two studies, variation in speech planning has received very little attention compared to the investigation of how much speakers encode before speaking.

This review of the literature focusing on experimental priming paradigms in the study of advance planning in the production of NPs is only shedding light on the many divergences remaining from a methodological and a theoretical point of view. The results of studies using phonological priming paradigms in the production of several words vary from facilitation effects limited to the first full word (Meyer, 1996; Schriefers and Teruel, 1999a,b see also Miozzo and Caramazza, 1999) to effects extending to the second word (Miozzo and Caramazza, 1999; Alario and Caramazza, 2002; Costa and Caramazza, 2002) or even the third word of a sequence (Schnur et al., 2006; Oppermann et al., 2010; Schnur, 2011). Moreover, whereas phonologically related primes usually facilitate the encoding of the related word by speeding up production latencies, several studies have reported interfering effects of phonologically related primes (Meyer, 1996; Jescheniak et al., 2003; Oppermann et al., 2010; Damian et al., under revision). Although there is not a very clear pattern arising from these results whether we group them according to languages (Germanic vs. Romance), the grammatical structure of the utterance tested or even the paradigm chosen, some trends emerge from the different studies. It seems indeed that it is more difficult to obtain a strong priming effect beyond the first word for Romance languages such as French (Dumay et al., 1999; Schriefers and Teruel, 1999a; Damian et al., under revision) and Italian (Miozzo and Caramazza, 1999). Only one study by Costa and Caramazza (2002) reports a priming effect for the second word in Spanish. While studies on English and German (Schnur et al., 2006; Damian and Dumay, 2007; Dumay et al., 2009; Oppermann et al., 2010; Schnur, 2011) very often report a span of encoding comprising the entire message, from simple NPs to verbal sentences. Only one study by Schriefers and Teruel (1999a,b) failed to report an effect on N in A+N sequences in German.

To try and account for these diverging results, we integrated two novel dimensions to the investigation of the span of phonological encoding in NPs in a Romance language. First, Experiment 1 examined advance phonological planning in NPs in French including the structure which has usually been investigated in Romance languages (N+A sequences), but also A+N sequences, which have not been investigated previously in a Romance language. Second, we explore inter-individual variability linked to production speed. Experiment 1 was a picture naming task with distractors phonologically related to the first or second word of the NP. The main effects obtained were a facilitation of primes related to both the noun (N) and the adjective (A) but only as an initial word in A+N and N+A sequences. Post-hoc analyses of these data suggested an interaction of speed with priming effects on the second word which suggests inter-individual differences in phonological planning. We investigated this hypothesis further in Experiment 2 on a larger group and showed that only slow speakers are facilitated by a phonological prime on the second word in adjectival NPs.

## Experiment 1

In this experiment, we investigated advance phonological planning in two different two-word French adjective-NPs in a PWI paradigm. Here participants produced NPs composed of a noun and an adjective appearing in two different syntactic orders: one with a pre-nominal adjective A+N (*grand chat*) and one with a post-nominal adjective N+A (*chat rouge*). Phonological distractors primed both the nouns and the adjectives.

If only the first word of French NPs is encoded at the phonological level as reported in previous studies, then we should observe a facilitation effect for both the noun and the adjectives being in the first position (in N+A and A+N, respectively) and no effect when being in the second position. By contrast, if previous cross-linguistic differences were due to the structure of adjectival NPs, then we should observe differences between the two types of NPs.

### Methods

#### Participants

Thirty French-speaking undergraduate students took part in the experiment. They received course credit for their participation.

#### Materials

In order to create forty adjective-NPs, we selected twenty disyllabic nouns and their corresponding pictures from a French database (Alario and Ferrand, 1999) with two pre-nominal and two post-nominal adjectives. Even though both pre-nominal and post-nominal adjectives exist in French, different adjectives “prefer” (Thuilier et al., 2012) one or the other position. For example, phonological length, frequency of the noun and of the adjective, noun-adjective semantic relationship within an NP has been described by some authors as being a good predictor of the position of the adjective (Waugh, 1977; Bouchard, 1998). The selected adjectives were either pre-nominal (*vieux* and *grand*) or post-nominal (*vert* and *rouge*) and their preferred position was respected, as *vieux* and *grand* are mostly used pre-nominally and color adjectives (*vert* and *rouge*) are always post-nominal (Thuilier et al., 2012). Each noun was associated with *vieux* or *grand* to create 20 adjective + noun NPs and with *vert* or *rouge* to form 20 noun + adjective NPs. To make sure that all sequences were used in French, we applied the method proposed by Blair et al.(2002, see also Janssen and Barber, 2012) based on Google counts: the frequency of the NP sequences were checked in the “French-speaking” Google web pages. (See Table A1 for stimuli details). In addition, the frequency of the NPs were similar across A+N and N+A sequences (*p* = 0.3).

For the A+N stimuli, the line drawings were colored in either red or green. For the N+A stimuli, the pictures were stretched to a larger size for the *grand* condition while they were depixelized in their normal size for the *vieux* condition.

Each noun and each adjective were associated with a phonological and an unrelated distractor from the same grammatical category. Twenty phonological distractors were disyllabic nouns sharing at least the first syllable (e.g., *balai* (broom) was primed by *ballon* (ball). In addition 20 unrelated disyllabic nouns [e.g., *commode* (drawer) for *balai*] were selected for the unrelated condition. They were not related semantically and did not share any phoneme with the target word. In order to reduce repetitions, two primes were selected for each adjective in each condition. Phonologically related primes shared the onset and at least an extra phoneme with the target adjectives. So for instance *vieux* (old) was primed once by *vide* (empty) and once by *vil* (vile) for the phonologically related condition while it was primed once by *chaud* (hot) and once by *doux* (soft) in the unrelated condition. The distractors were presented auditorily.

#### Procedure

Before the experiment, participants were familiarized with all the pictures and their corresponding nouns and adjectives on a paper sheet. The stimuli appeared on a computer screen and participants were instructed to name them aloud with the corresponding NP as quickly and as accurately as possible and to ignore the words they heard in the headphones. A short training session with filler items preceded the experimental session and was repeated if necessary until the subjects felt confident about the instructions. Stimulus presentation was controlled by the DMDX software (Forster and Forster, 2003). Each trial had the following structure: fixation cross stayed on the screen for 500 ms, followed by a 200 ms blank screen, then the stimulus (the picture) appeared on the screen at the same time as the distractor word played in the headphones (at SOA 0). The picture remained on the screen for 3000 ms. A blank screen followed and stayed for 2000 ms before the next trial.

Each stimulus appeared once in each condition (i.e., with phonologically related or unrelated prime to the adjective or to the noun). The order of presentation of the 160 stimuli was pseudo-randomized in four blocks so that each stimulus appeared once in each block and blocks were counterbalanced across participants. There was a pause every two blocks. Production latencies (RTs) were measured starting from the onset of the picture to the onset of the vocal response.

### Results

The measurement of naming latencies was operated by means of a voice key. Voice key failures to detect the acoustic onset of the target word were systematically checked and corrected with speech analyser software. Errors, no responses and technical errors were discarded from the analysis. As mixed models were used for the data analysis, only extreme outliers (reaction times above 1700 and below 400 ms) and not standard deviations were withdrawn from the data analysis following Baayen and Milin's (2010) recommendation. A total of 14% of the RT data was removed. The results are presented in Table 1.

**Table 1:** **Mean RTs in ms (SD in brackets) and error rate for each condition at SOA 0 (Experiment 1)**.

	**NP**	**Mean (*SD*)**		**Difference (ms)**	**Error (%)**	
		**Phonologically related**	**Unrelated**		**Phonologically related**	**Unrelated**
Word 1 primed	**A**+N	774 (168)	787 (175)	13^*^	1.5	1.8
	**N**+A	855 (203)	871 (209)	16^*^	1.8	1.7
Word 2 primed	A+**N**	798 (177)	807 (192)	9	1.7	1.7
	N+**A**	860 (196)	852 (193)	−8	1.7	1.9

* *Refers to the values which reach statistical significance (*p* > *0.05*). Bold letters refer to the words which are primed by a phonological distractor*.

Spoken latencies data were fitted with linear regression mixed models (Baayen et al., 2008) with the R-software (R-project, R Development Core Team, 2005; Bates and Sarkar, 2007). We analyzed the two datasets separately according to the position of the element related to the prime: the first elements, whether it was the adjectives or the noun (W1 priming) and the second elements (W2 priming).

The syntactic order (A+N, N+A) and distractors (unrelated, phonologically related) were included in linear mixed-effects models as fixed effect variables with participants and items as random effect variables. The more complex variance structure with by-participant and by-item adjustments on both slopes and intercept were included in the model as recommended by Barr et al. (2013) who argue that the inclusion of condition-specific random effects by subjects/items for every fixed effect of theoretical interest that is measured in more than one condition within subjects/items reduces the chances of obtaining Type I errors. Error rates were fitted with logit mixed-effects models (Jaeger, 2008) with the same random- and fixed-effects factors.

For W1 priming, the facilitation effect of the distractor condition was significant [*t*_(2052)_ = 2.00; *p* < 0.01] without interaction between priming and syntactic order (*t* < 1). We also observed an effect of the syntactic order condition [*t*_(2052)_ = 8.6; *p* < 0.0001] on RTs with A+N sequences being produced faster than N+A sequences. The error rate did not differ between the phonologically related condition and the neutral condition (*z* < 1) for the W1 priming nor for the order condition (*z* < 1).

For W2 priming, there was no effect of the distractor: (*t* < 1) and no interaction between priming and syntactic order (*t* < 1). The only significant effect observed was the syntactic order effect [*t*_(2048)_ = 5.47; *p* < 0.004], with shorter latencies for A+N than for N+A.

The error rate analysis did not differ across conditions (all *z* < 1).

### Discussion

Results from Experiment 1 suggest that phonological priming effects are limited to the first word of adjective-NPs, whether it is an adjective or a noun. These results seem to indicate that only the first element of the NP is encoded at the phonological level no matter the syntactical status or the order of the constituents. Overall, these findings are in line with previous results reporting phonological priming limited to the first word of the sentence (Meyer, 1996; Miozzo and Caramazza, 1999; Schriefers and Teruel, 1999a,b; Damian et al., under revision) but not with those reporting a larger encoding span (Costa and Caramazza, 2002; Schnur et al., 2006; Schnur, 2011). In particular, the present results are congruent with previous studies on post-nominal adjectival NPs reporting an effect of priming limited to the N in French (Schriefers and Teruel, 1999a; Dumay et al., 2009; Damian et al., under revision). By contrast, the lack of phonological priming effects on the second word in A+N sequences is in contradiction with several previous studies reporting a priming effect on N, although in other languages (Costa and Caramazza, 2002 in English; Dumay et al., 2009 in English).

**Figure 1:**
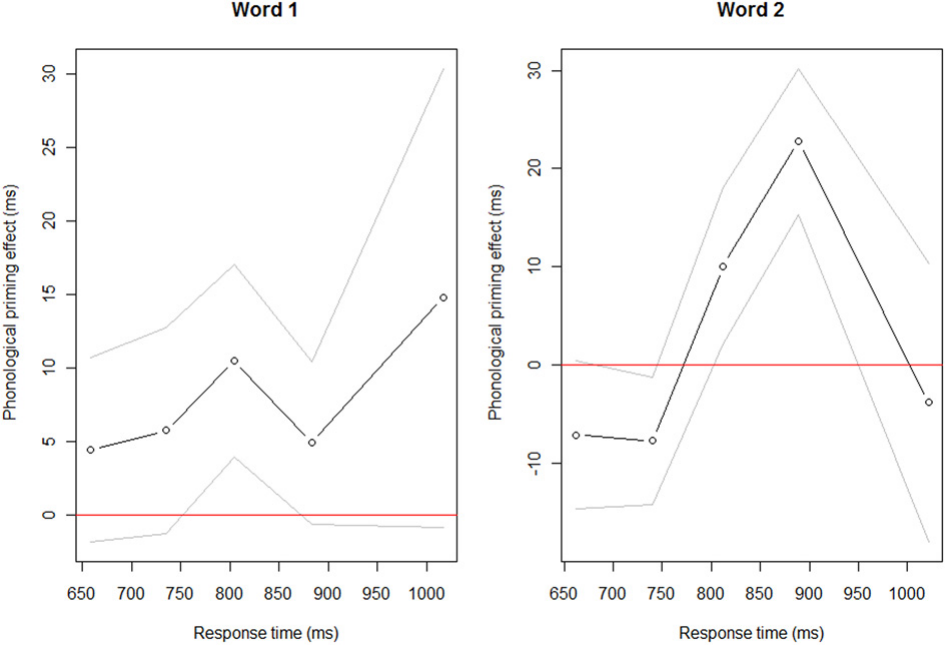
**Delta plots for the priming effect (phonologically related or unrelated) of the first word of the NP and the second word of the NP respectively at a neutral SOA**. On the x-axis is the distribution of naming latencies. On the y-axis is the size of the effect (positive values represent the facilitation effect while negative values represent an inhibitory effect). The distribution of the RTs is averaged per quantile (here five quantiles represented by the circles on the plot) and participants.

Along with the arguments in favor of the encoding up to the N in prenominal adjectival NPs outlined in the literature, the lack of significant priming effect on the second word may be due to the fact that the span of encoding varies. As suggested by Wagner et al. (2010) and Ferreira and Swets (2002), speakers might use different encoding strategies, in particular in experimental tasks, leading to null results at the group level. This interpretation can be addressed by displaying the data of all participants in a so-called *delta plot* (De Jong et al., 1994). *Delta plots* allow us to display the phonological priming effect as a function of the distribution of the naming latencies of all the participants. This comparison is done by plotting the quantiles of one condition (i.e., the phonologically related condition) against the quantiles of another condition (i.e., the phonologically unrelated condition) and determine whether the two populations present a common distribution. *Delta plots* are expected to display the phonological priming effect as a positive slope if this effect is facilitatory. If, as we would like to argue, encoding of W2 (but not W1) is subject to variability as a function of speakers' naming latencies, we should observe a change of the effect across time in the *delta plot* for W2 but not W1. Figure 1 displays the priming effect for W1 and W2, respectively. The slope for the priming of W1 is positive and does not change as a function of speakers' naming latencies. The effect is consistent for all types of speakers. Contrastively, priming of W2 presents a different pattern. While fast naming latencies (RTs between 650 ms until approximately 800 ms) do not reveal a facilitation effect, a positive slope increases along with longer naming latencies (between approximately 800–950 ms) and decreases again with the slowest naming latencies. This plotting clearly shows that the effect varies as a function of speakers' naming latencies for priming of the second element of the NP only, and that no variation is observed for W1 priming.

This suggests that speakers' encoding of the second word varies across naming latencies and the amount of encoding beyond the initial word is not the same for all speakers.

In sum, results from Experiment 1 seem to indicate that phonological encoding processes are not determined by order in the production of French adjective NPs and that the syntactic status of the words located in the phonological frame does not modulate phonological planning. It seems that when producing NPs in French, speakers can start articulating their message as soon as the first phonological word is encoded and that the amount of advance planning can be smaller than the phrase.

Can we assume, based on this conclusion, that the span of phonological encoding in French NPs is limited to one phonological word? This assumption is perfectly coherent with previous accounts for N+A sequences: encoding of the N only in N+A NPs is in agreement not only with the literature (except for the cross-linguistic study by Costa and Caramazza, 2002) but also with Schriefers and Teruel's (1999a) smallest full syntactic phrase theory, according to which the head noun determines encoding processes at least at the lexical encoding level. However, encoding limited to the A in A+N NPs is challenging on several points. First, it is not coherent with the literature as all but one (Schriefers and Teruel, 1999b) studies reported a span of encoding extending the initial word in A+N NPs. Last but not least, it can hardly account for the production of obligatory *liaison* where planning is assumed to be necessary to produce that type of sequence correctly. The diverging results from the literature and the examination of results according to production latencies in Experiment 1 rather suggest that the span of encoding varies according to inter-individual encoding strategies. To investigate this hypothesis further, we focused on A+N NPs with the inclusion of sequences involving *liaison*. With regards to the literature, A+N NPs are most likely to present a larger span of encoding and be sensitive to, if there are any, differences in encoding strategies.

## Experiment 2

As underlined in the introduction, besides syntactic factors, variables linked to the subjects have also been assumed to modulate the amount of advance planning: Wagner et al. (2010) and Gillespie and Pearlmutter (2011) reported that speakers with slower speech onset latencies presented a larger span of encoding than speakers with longer latencies. To investigate whether the lack of facilitation effect on the second word in A+N NPs in Experiment 1 can be explained by possible individual differences in speech planning, the speakers of Experiment 2 were divided into two “speed” sub-groups according to their mean RTs. Based on the Wagner et al. (2010) and Gillespie and Pearlmutter (2011) studies, we expect to find a facilitation effect on the second word in A+N sequences for speakers with slower onset latencies only. Thus, failure to obtain an effect on N in A+N sequences in Experiment 1 may be related to speakers' initiation strategies. We tested a larger group of speakers which could be split into sub-groups according to initialization speed as was done in Gillespie and Pearlmutter's study. In addition, to make sure that our participants behaved in an experimental task as they would in a natural speech context, we selected them according to their ability to produce the obligatory *liaison* correctly in the experimental paradigm. While French speakers very seldom fail to produce a *liaison* in a natural context, we indeed noticed that some participants surprisingly tended to do so in experimental paradigms. We included ¼ of obligatory *liaison* sequences in our material in order to exclude subjects who would display a rare production pattern in the experimental paradigms, i.e., the omission of obligatory *liaison* consonants. As mentioned in the Introduction, the French *liaison* involves both syntactic and phonological constraints which imply a larger span of encoding at least up to the phonological encoding level. Two conditions need indeed to be met in the correct production of a *liaison* in French. On a phonological level, a final latent consonant of a word becomes realized when followed by a vowel-initial word (e.g., /gRã ami becomes/gRã tami/). On a syntactic level, *liaison* is obligatory only in certain types of syntactic structures, namely A+N NPs but not N+A NPs (Stark and Pomino, 2009). The omission of *liaison* consonants would indicate that subjects do not encode NP sequences in an experimental setting in the same way they would encode it in natural speech. Results from Experiment 2 should therefore provide us with more information on whether speech latencies affect phonological encoding processes and on whether participants employ rare encoding strategies in this kind of experimental paradigms.

### Methods

#### Participants

Sixty-one French speaking undergraduate students of the University of Geneva took part in the experiment. They received course credit for their participation. All had normal or corrected-to-normal vision.

#### Materials

**Figure 2:**
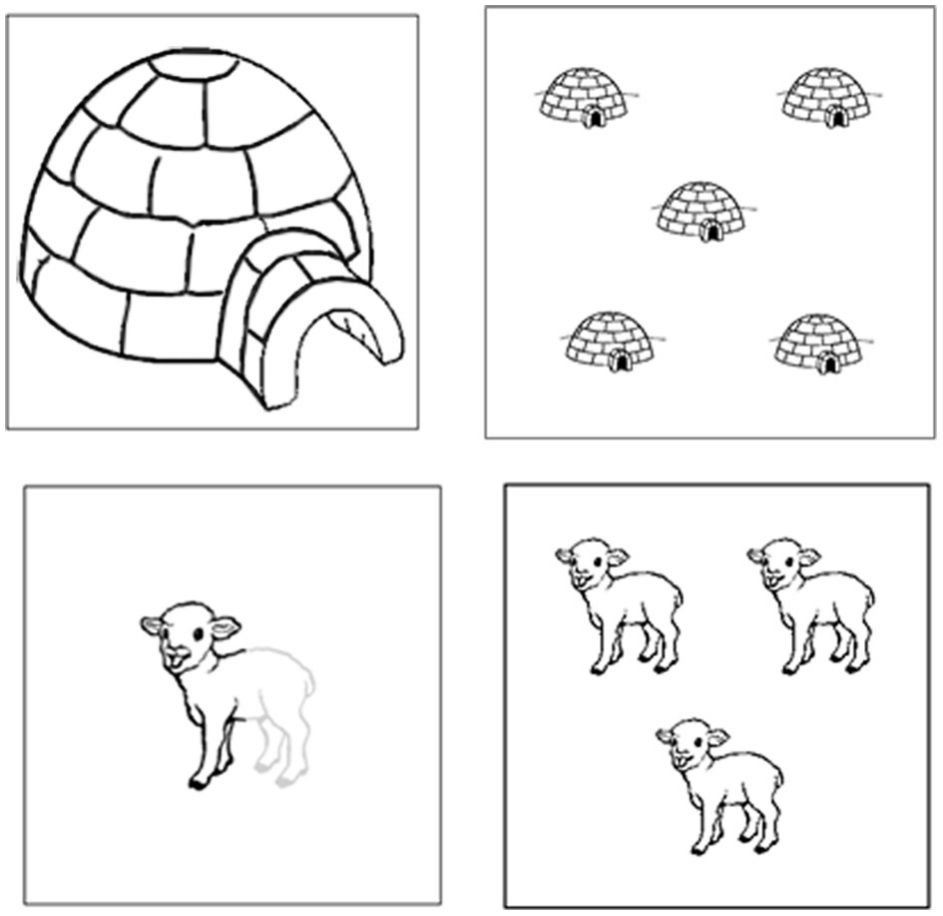
**Illustration of the four different conditions in Experiment 2 with (clockwise): “le grand igloo (the big igloo), les cinq igloos (the five igloos), le demi agneau (the half lamb), les trois agneaux (the three lambs)**.”

Twelve disyllabic nouns and their corresponding pictures were selected from the French database by Alario and Ferrand (1999). (See characteristics in Table A2). Half of the nouns started with a vowel and the other half with a consonant. Four adjectives were selected. Two of them required an obligatory *liaison* when followed by a vowel-initial noun [*trois* (three) and *grand* (big)] while the two others did not involve any external sandhi phenomenon: *demi* (half) and *vieux* (old). A quarter of the sequences involved an obligatory *liaison* between A and N (e.g., *les trois aimants*, “the three magnets”). Examples of the stimuli are presented in Figure 2. As in Experiment 1, each NP was associated with a distractor which was either phonologically related or unrelated to the target noun or adjective. Each noun was associated with one of the two types of adjectives and each sequence appeared four times: primes related phonologically or unrelated to the noun and to the adjective. Four additional nouns were associated with the four adjectives and used for training items.

#### Procedure

The procedure was exactly the same as in Experiment 1, with auditory distractors presented at SOA 0. Each participant produced a total of 96 NPs in the experimental part preceded by a training session on 16 filler trials. The NPs were presented in pseudo-randomized order in four blocks which were counterbalanced across participants.

### Results

Voice key failures were checked and corrected with speech analyser software. Errors, no responses and technical RT errors were discarded from the analysis. Extreme reaction times above 1300 and below 320 ms were withdrawn from the data analysis (Baayen and Milin, 2010). A total of 7% of the RTs was therefore removed. Three participants were removed because of high error rate + high *liaison* error rate (25% or more on the total of the 96 NPs). Based on the hypothesis that the correct production of a *liaison* sequence requires advance planning, we categorized 20 participants who omitted the *liaison* consonant at least twice (i.e., on more than 8% of the NPs involving obligatory *liaison*) as a sub-group which will be analyzed separately. The reason for this strict criterion is because French speakers, in a natural speech context, would rarely neglect to produce such obligatory *liaisons*. This suggests that failure to produce the *liaison* might reflect inter-individual strategies in an experimental context.

The 38 remaining participants were divided into two sub-groups according to their average naming latencies. A group of 19 speakers constituted the “slow” sub-group (mean latencies: 795 ms) and the remaining 19 the “fast” sub-group (mean latencies: 556 ms).

Spoken latencies data were fitted with linear regression mixed models (Baayen et al., 2008) with the R-software (R-project, R Development Core Team, 2005; Bates and Sarkar, 2007).

As in Experiment 1, we first separated the data into two datasets: the data where the first elements were primed (the adjectives) and in the data were the second elements that were primed (the nouns). The speed (fast, slow) and distractor (unrelated, phonologically related) were included in a general linear mixed-effects model as a fixed effect variable and participants and items as random effect variables. The more complex variance structure (random-intercept and random-slopes) was included. Error rates were fitted with logit mixed-effects models (Jaeger, 2008) with same random- and fixed-effects factors.

### Priming of the adjective (W1)

The results are presented in Table 2.

**Table 2:** **Mean RTs in ms (SD in brackets) and error rate for each condition for W1 priming (Experiment 2)**.

**Speed**	**Mean (*SD*)**		**Difference (ms)**	**Errors (%)**	
	**Phonologically related**	**Unrelated**		**Phonologically related**	**Unrelated**
Fast	570 (84)	544 (72)	−26^*^	0.9	0.8
Slow	802 (116)	769 (104)	−33^*^	1.2	1.8
Total	686 (100)	656 (88)	−30	2.2	1.6

* *Refers to the values which reach statistical significance (*p* > *0.05*)*.

We observed a significant effect of interference [*t*_(1621)_ = 4.350; *p* < 0.0001] with longer naming latencies for the phonologically related condition (686 ms) relative to the unrelated condition (656 ms) with an effect of the speed [*t*_(1621)_ = −6.952, *p* < 0.0001] but no interaction between speed and priming (*t* < 1). The error rate did not differ significantly between the phonologically related condition and the unrelated condition (*z* < 1), nor between speed sub-groups and there was no interaction between the priming and speed sub-groups.

### Priming of the noun (W2)

The results are presented in Table 3. A main effect of priming was observed: [*t*_(1598)_ = −4.041, *p* < 0.0001] and an interaction between speed sub-groups and priming: [*t*_(1598)_ = 2.715; *p* < 0.0012]. Contrasts between the two speed sub-groups showed that priming was not significant for the fast speakers (*t* < 1) while the priming effect was significant for the slow speakers: [*t*_(759)_ = −3.54; *p* < 0.0002] with faster naming latencies for the phonological condition (790 ms) relative to the unrelated condition (820 ms). The error rate analysis indicated no significant difference between the phonologically related condition and the unrelated condition (*z* < 1), a main effect of speed (*z* = −2.708, *p* < 0.006) with a higher error rate for the slow speakers, and no interaction between the priming condition and the speed sub-groups.

**Table 3:** **Mean RTs in ms (SD in brackets) and error rate for each condition for W2 priming (Experiment 2)**.

**Speed**	**Mean (*SD*)**		**Difference (ms)**	**Errors (%)**	
	**Phonologically related**	**Unrelated**		**Phonologically related**	**Unrelated**
Fast	556 (82)	557 (79)	2	0.9	0.6
Slow	790 (106)	820 (116)	30^*^	1.5	1.5
Total	673 (94)	689 (98)	16	2.4	2.1

* *Refers to the values which reach statistical significance (*p* > *0.05*)*.

### Discussion

The aim of this experiment was to investigate variation of phonological planning due to inter-individual strategies and to explore whether phonological encoding of French NPs could extend beyond the initial word. To this aim we only retained among our participants those who produced obligatory *liaison* sequences correctly to make sure that the group of participants we tested did, in theory, behave in the experimental task as they would in more natural conditions. Furthermore, we analyzed separately participants with short and long mean production latencies. Results revealed that as far as phonological encoding of the first word of a NP is concerned, the same inhibitory effects are observed for the two speed sub-groups of participants (fast or slow). Contrary to the results reported for the adjectives, analyses of the N in A+N revealed priming of the noun limited to the group of slow speakers. To support these results, we ran additional correlational analyses between the size of the priming effect and the speed of all participants for W1 and W2, respectively. A significant positive correlation was observed for W2 only [*r*_(36)_ = 0.34, *p* < 0.033] but not for W1 (*p* = 0.31) indicating that the priming effect for W2 increases with an increase in production latencies.

Furthermore, even if we did not include them in the main analysis, we must mention the sub-group of 20 participants who failed to produce *liaison* sequences correctly. If we consider that *liaison* is an indicator of advance planning, then we suggest that those speakers who did not produce *liaison* sequences correctly might present a span of encoding limited to the initial word. Post hoc analysis does indeed show a lack of priming effect on the N (*t* < 1) for these speakers. These speakers have rather fast mean production latencies (637 ms) and probably do not produce the *liaison* in an experimental setting because they plan word by word. We will further discuss this result in the general discussion.

Another result merits to be discussed here. While both Experiments 1 and 2 present a phonological priming effect on the first word of the sequences, it is worth noting that the priming effect on the adjective in Experiment 2 is inhibitory, whereas it is facilitatory in Experiment1 for similar NPs. Even though the focus of this work is on advance planning beyond the initial word (the N in A+N), we ought to discuss the diverging polarity of this effect across our experiments. Despite the fact that the two experiments presented similar designs, a few differences may account for the opposite patterns of the priming effect.

First, two different words were used to prime each adjective in the A+N condition in Experiment 1 (four distractor adjectives primed two pre-nominal adjectives in total) while a single adjective was used to prime each adjective in Experiment 2 (four distractor adjectives primed four adjectives in total). Crucially, all the primes in Experiment 2 were strictly post-nominal adjectives and the target adjectives were all pre-nominal adjective, while half of the primes in Experiment 1 were pre-nominal as the target adjective. This difference in the syntactic structure of the prime relative to the target might have led to a conflict at the syntactic level in Experiment 2, leading to interference, whereas this conflict might be weaker in Experiment 1, allowing phonological facilitation to emerge. Moreover, Experiment 1 presented two different syntactic structures (A+N and N+A) while Experiment 2 only presented A+N NPs. This repetition of the same syntactic structure might have led to some syntactic priming of the A+N sequence in Experiment 2 compared to Experiment 1 where syntactic priming was prevented by the use of two different syntactic structures. The fact that repetition of A+N sequences might have led to syntactic priming in Experiment 2, and the fact that pre-nominal distractors were primed with post-nominal distractors, are converging arguments to suggest interference at the syntactic level preventing the facilitation effect from emerging. All in all, the fact that a difference is observed between the two conditions, independently of the polarity of the effect, shows that there must have been activation of the phonological form of the critical word before speech initiates.

Taken on its own, this experiment suggests that inter-subject variability can account for different encoding patterns at the level of phonological encoding in a picture naming task. This result is in line with results on advance planning at the grammatical level (Wagner et al., 2010) reporting different patterns for fast and slow subjects. In addition, the present experiment also indicates that a high proportion of speakers (30%) seem to adopt unusual speech encoding strategies while performing experimental tasks, as suggested by the rates of omission of *liaison* consonants in obligatory contexts. This observation calls into question the reliability of the interpretation of data collected by this kind of experimental paradigm as also underlined by other authors (Jaeger et al., 2012). These results could explain why Schriefers and Teruel (1999b) failed to observe a priming effect on the N in A+N in their study while most studies report a priming effect for the entire A+N NP.

## General discussion

The question of how much speakers plan ahead before they start articulating is very complex to address experimentally: phonological advance planning in NPs has been investigated in several languages, with different experimental paradigms and several incoherent results appearing in the literature. The present study investigated whether inter-subject variability can account for the diverging results on the span of phonological encoding of NPs in French.

The first experiment investigated phonological advance planning in French NPs with a PWI paradigm and included for the first time pre-nominal adjectives in a Romance language. The results of Experiment 1 revealed that the first element of the NP was primed by a phonologically related distractor independently of its grammatical category (noun or adjective) and independently of the order of its constituents (A+N or N+A). By contrast, no priming effect was observed when the second word was primed. *Delta plot* displays of the data suggested modulation of phonological priming effects by speed of initialization. We further investigated the inter-subject variability hypothesis in Experiment 2. Results clearly showed that slow and fast participants presented different phonological priming patterns on the last element of the NP; while the first word was inhibited by a phonologically related word for all speakers, only the slow speaker group presented a priming effect on the second element of the NP. Additional correlational analyses supported this pattern of results as a significant correlation between the size of the priming effect and the speed of participants was reported for the second element of the NPs only.

Thus, for slow initializing subjects, we observed a priming effect on the second element of adjective-NPs with prenominal adjectives. This structure has not been tested previously in a Romance language, where only post-nominal adjectives have been considered so far. Our results on A+N sequences for slow speakers are in agreement with most results from studies investigating this type of structure (A+N) in Germanic languages where it represents the dominant structure (Schriefers and Teruel, 1999a; Dumay et al., 2009; Damian et al., under revision). Whereas it is plausible that phonological encoding is limited to the initial word in N+A sequences as reported in most studies in Romance languages (Schriefers and Teruel, 1999a; Dumay et al., 2009; Damian et al. under revision), encoding of the adjective only in A+N seems less likely since the adjective does not represent a full syntactic phrase (Schriefers and Teruel, 1999a). Moreover, according to some authors (Kuipers and La Heij, 2009; Dumay and Damian, 2011) the noun should receive automatic activation from being the “object” of the NP while the adjective being only an “attribute” will not. Then priming on the noun in A+N sequences should ease the encoding of the sequence. Only the results of Schriefers and Teruel (1999b) in German A+N sequences and our results with fast speakers fail to converge with an encoding of the entire NP in the case of prenominal adjectives. It is possible that the behavior of the group of speakers in Schriefers and Teruel's (1999b) study was similar to our fast group.

### Speakers' strategies

An additional issue with results pointing to encoding of the adjective only in A+N is related to the production of specific sandhi phenomena such as the French *liaison* which is obligatory in such sequences. The inclusion of sequences involving obligatory *liaison* in Experiment 2 allowed us to identify a number of participants who failed to produce the *liaison*. This observation suggests that participants use specific encoding strategies in experimental settings which they would not apply in natural settings. Therefore, two sources of variability linked to the participants have been identified in Experiment 2. Whereas the omission of obligatory *liaison* indicates that those speakers adopt specific speech planning strategies in experimental settings, it is unclear whether the source of variability among speakers with fast or slow initialization is linked exclusively to speakers' behavior in experimental sessions or if it reflects their usual behavior. Only speakers with long production latencies showed a priming effect on the second element of the NPs, while fast speakers seemed to articulate once the phonological code of the first word was available. Similar variations have already been reported by Gillespie and Pearlmutter (2011) and Wagner et al. (2010). In experimental contexts, speakers are often instructed to name the pictures as fast and as accurately as possible. Because the right balance between the two is not easy to find, some speakers might favor time and initiate speech as soon as one word is encoded while others might favor preparation of the entire message.

### Speakers' variability

The analysis according to production speed in Experiment 1 clearly showed that the priming effect was modulated as a function of participants' reaction times. Although a U-shape tendency was observed, which was not in favor of a clear-cut distinction of speech initialization, we analyzed the two speed sub-groups similarly to the strategy adopted in previous studies (Gillespie and Pearlmutter, 2011 and Wagner et al., 2010) in Experiment 2. As there is very little input on the topic of between-subject variability, and because no other significant criterion has been reported in the psycholinguistic literature to our knowledge, we opted for the same distinction (slow and fast speakers). Nevertheless, while some authors argue that speed of initialization modulates speech planning, we would like to argue that the fact that some speakers present a larger span of encoding probably leads to a delay in speech initialization. So instead of claiming that slow speakers present a larger span of encoding, we claim that speakers with a large span of encoding start articulating their message later. These speakers are not “slow speakers” but speakers with a larger planning unit and therefore “slow initializing” speakers.

Taken together, the distribution of the priming effect on the second word, its interaction with speed of initialization and the omission to produce obligatory *liaison* in some speakers are clear indicators of inter-individual differences among participants in an experimental task.

The overall pattern of results in Experiment 1 and the results for the fast initializing group in Experiment 2 are in line with a word-by-word incremental view of speech planning. However, results from slow initializing speakers indicate that the minimal amount of encoding can extend the initial word.

Overall these results favor the hypothesis that speech is not strictly incremental but under strategic control (Ferreira and Swets, 2002; Ferreira and Engelhardt, 2006; Konopka, 2012). It is however also possible that the syntactic structure drives phonological encoding processes as a default process but that other external constraints (time pressure, overcorrection, stress etc.) can overrule this default program, as claimed by Martin et al. (2010). In other words, if the production context presents no specific focus, phonological encoding processes may be determined by syntactic structure. In which case, the first smallest full syntactic phrase would specify the amount of advance planning. However, if the production context requires specific encoding modalities (as, for instance, in an experimental paradigm), then speakers might modulate their encoding strategies. While our results are additional evidence for speaker's variability in phonological planning, they do not allow us to suggest which factors might modulate the span of encoding.

## Conclusion

The diverging results reported in the literature on advance phonological planning may partly be reconciled in light of the present results where some speakers seem to encode word-by-word whereas others encode beyond the first phonological word. Crucially, this study underlines the need to focus on which variables constrain the span of phonological encoding rather than on how much is encoded before articulation, as there does not seem to be a fixed amount of advance planning.

### Conflict of interest statement

The authors declare that the research was conducted in the absence of any commercial or financial relationships that could be construed as a potential conflict of interest.

## Appendix

**Table A1:** **Adjectival NPs and their internet frequency in Experiment 1 and phonologically related and unrelated noun distractors**.

**Noun (N)**	**Internet NP frequency**				**Distractors**	
	**Post-nominal A (N+A)**		**Pre-nominal A (A+N)**		**Phonological distractors**	**Unrelated distractors**
	***Vert* (green)**	***Rouge* (red)**	***Grand* (Big)**	***Vieux* (old)**		
Balai (broom)		868000		609000	Ballon (balloon)	Commode (drawer)
Cadenas (locker)		597000	2030000		Cadeau (gift)	Souris (mouse)
Canard (Duck)		7269000	1770000		Cafard (cockroach)	Etoile (star)
Chapeau (hat)	1680000			2120000	Château (castle)	Fougère (fern)
Citron (lemon)		1230000	1900000		Siphon (siphon)	Fourchette (fork)
Cochon (pig)	559000		3780000		Coton (cotton)	Pastèque (watermelon)
Croissant (croissant)	1420000			1150000	Croyant (believer)	Horloge (clock)
Gâteau (cake)		957000	2390000		Garrot (tourniquet)	Maison (house)
Maïs (corn)		29500000	172000000		Masseur (masseur)	Bouteille (bottle)
Palmier (palm tree)	428000		775000		Palier (langing)	Tortue (turtle)
Pinceau (brush)		840000		328000	Pincer (pinch)	Tomate (tomato)
Poisson (fish)	901000		307000		Poison (poison)	Cravate (tie)
Raisin (grapes)		1170000	2770000		Réseau (network)	Valise (suitcase)
Renard (fox)		880000		663000	Retard (delay)	Echelle (ladder)
Serpent (snake)	717000		1140000		Serment (oath)	Chemise (shirt)
Soleil (sun)	780000			5580000	Sommeil (sleep)	Poupée (doll)
Stylo (pen)	600000			435000	Styliste (stylist)	Trompette (trumpet)
Tonneau (barrel)		515000		294000	Tonnerre (thunder)	Fenêtre (window)
Tracteur (tractor)	606000			254000	Trappeur (trapper)	Enveloppe (envelope)
Vélo (bike)		1980000		1730000	Véto (veto)	Fourmi (ant)

*Each noun was associated with only one of the two adjectives in each condition (A+N and N+A)*.

**Table A2:** **Noun stimuli and distractors in Experiment 2**.

**Adjective**	**Target noun**	**Phonological distractors**	**Unrelated distractors**
Demi/trois	Agneau	Habit	Butin
(half)/(three)	(lamb)	(clothes)	(booty)
Demi/trois	Aimant	Été	Moulin
(half)/(three)	(magnet)	(summer)	(mill)
Cinq/grand	Avion	Appui	Mari
(five)/(big)	(plane)	(support)	(husband)
Cinq/grand	Cactus	Castor	Dormeur
(five)/(big)	(cactus)	(beaver)	(sleeper)
Cinq/grand	Camion	Casier	Media
(five)/(big)	(lorry)	(locker)	(media)
Demi/trois	Citron	Sigma	Respect
(half)/(three)	(lemon)	(sigma)	(respect)
Cinq/grand	Eclair	Effluve	Facteur
(five)/(big)	(lightning)	(effluvium)	(postman)
Demi/trois	Gâteau	Galet	Debut
(half)/(three)	(cake)	(pebble)	(start)
Cinq/grand	Igloo	Iguane	Bougeoir
(five)/(big)	(igloo)	(iguana)	(candlestick)
Demi/trois	Indien	Impôt	Fagot
(half)/(three)	(Indian)	(taxes)	(bundle)
Demi/trois	Panier	Patio	Convoi
(half)/(three)	(basket)	(patio)	(convoy)
Cinq/grand	Pingouin	Pinceau	Muguet
(five)/(big)	(pinguin)	(brush)	(lily)

## References

[B1] Alario F. X. Caramazza A. (2002). The production of determiners: evidence from French. Cognition 82, 179–223 10.1016/S0010-0277(01)00158-5 11747862

[B2] Alario F. X. Costa A. Caramazza A. (2002). Frequency effects in noun phrase production: Implications for models of lexical access. Lang. Cogn. Process. 17, 299–319 10.1080/01690960143000236

[B3] Alario F. X. Ferrand L. (1999). A set of 400 pictures standardized for French: Norms for name agreement, image agreement, familiarity visual complexity, image variability, and age of acquisition. Behav. Res. Methods Inst. Comput. 31, 531–552 10.3758/BF03200732 10502875

[B4] Baayen R. H. Davidson D. Bates D. M. (2008). Mixed-effects modeling with crossed random effects for subjects and items. J. Mem. Lang. 59, 390–412 10.1016/j.jml.2007.12.005

[B5] Baayen R. H. Milin P. (2010). Analyzing reaction times. Int. J. Psychol. Res. 3.2, 12–28

[B6] Barr D. J. Levy R. Scheepers C. Tily H. (2013). Random-effects structure for confirmatory hypothesis testing: keep it maximal. J. Mem. Lang. 68, 255–278 10.1016/j.jml.2012.11.001 24403724 PMC3881361

[B7] Bates D. M. Sarkar D. (2007). Lmer4: Linear Mixed-Effects Models Using S4 Classes. R package version 0.99875–6.

[B8] Blair I. V. Urland G. R. Ma J. E. (2002). Using internet search engines to estimate word frequency. Behav. Res. Methods Inst. Comput. 34, 286–290 10.3758/BF03195456 12109025

[B9] Bouchard D. (1998). The distribution and interpretation of adjectives in French: a consequence of bare phrase structure. Probus 10, 139–183 10.1515/prbs.1998.10.2.139

[B10] Bybee J. (2001). Frequency effects on French Liaison in Frequency and the Emergence of Linguistic Structure, eds Joan Bybee Paul Hopper (Amsterdam: John Benjamins), 337–359 10.1075/tsl.45

[B11] Costa A. Caramazza A. (2002). The production of noun phrases in English and Spanish: Implications for the scope of phonological encoding in speech production. J. Mem. Lang. 46, 178–198 10.1006/jmla.2001.2804

[B12] Damian M. F. Dumay N. (2007). Effects of phoneme repetition in spoken utterance generation in Proceedings of the 16th International Congress of Phonetic Sciences, eds Trouvain J. Barry W. J. (Saarbrücken), 589–592

[B14] De Jong R. Liang C. C. Lauber E. (1994). Conditional and unconditional automaticity: a dual-process model of effects of spatial stimulus-response concordance. J. Exp. Psychol. Hum. Percept. Perform. 20, 731–750 10.1037/0096-1523.20.4.731 8083631

[B15] Dell G. (1986). A spreading-activation theory of retrieval in sentence production. Psychol. Rev. 93, 283–321 10.1037/0033-295X.93.3.283 3749399

[B15a] Dumay N. Content A. Frauenfelder U. H. (1999). Acoustic-phonetic cues to word boundary location: evidence from word spotting in Proceedings of the 14th International Congress of Phonetic Sciences (San Francisco, CA), 281–284

[B15b] Dumay N. Damian M. F. (2011). A word order constraint in single word production? Failure to replicate Janssen, Alario and Caramazza (2008). Psychol. Sci. 22, 559–561 10.1177/0956797611401754 21389337

[B16] Dumay N. Damian M. F. Stadthagen-Gonzalez H. Perez M. A. (2009). Is the scope of phonological encoding constrained by the syntactic role of the utterance constituents? in Proceedings of the 31st Annual Conference of the Cognitive Science Society, eds Taatgen N. A. van Rijn H. (Austin, TX: Cognitive Science Society), 667–672

[B17] Encrevé P. (1988). La liaison Avec et sans Enchaînement. Phonologie Tridimensionnelle et Usages du Français. Paris: Le Seuil

[B19] Ferreira F. Engelhardt P. (2006). Syntax and production in Handbook of Psycholinguistics, eds Gernsbacher M. A. Traxler M. (Oxford: Elsevier Inc), 61–91 10.1016/B978-012369374-7/50004-3

[B20] Ferreira F. Swets B. (2002). How incremental is language production? Evidence from the production of utterances requiring the computation of arithmetic sums. J. Mem. Lang. 46, 57–84 10.1006/jmla.2001.2797

[B21] Forster K. L. Forster J. C. (2003). DMDX: a windows display program with millisecond accuracy. Behav. Res. Methods Inst. Comput. 35, 116–124 10.3758/BF03195503 12723786

[B22] Fromkin V. (ed.). (1973). Speech Errors as Linguistic Evidence. The Hague: Mouton

[B23] Garrett M. F. (1975). The analysis of sentence production in The Psychology of Learning and Motivation. Vol. 9: Advances in Research and Theory, ed Bower G. H. (New York, NY: Academic Press), 133–177

[B24] Garrett M. F. (1980). Levels of processing in sentence production in Language production: Vol. 1. Speech and Talk, ed Butterworth B. (London: Academic Press), 177–220

[B25] Gillespie M. Pearlmutter N. J. (2011). Effects of semantic integration and advance planning on grammatical encoding in sentence production in Proceedings of the 33rd Annual Conference of the Cognitive Science Society, eds Carlson L. Holscher C. Shipley T. (Austin, TX: Cognitive Science Society), 1625–1630

[B26] Jaeger T. F. (2008). Categorical data analysis: away from ANOVAs (transformation or not) and towards logit mixed models. J. Mem. Lang. 59, 434–446 10.1016/j.jml.2007.11.007 19884961 PMC2613284

[B27] Jaeger T. F. Furth K. Hilliard C. (2012). Phonological encoding during unscripted sentence production. Front. Psychol. 3:481 10.3389/fpsyg.2012.00481 23162515 PMC3497891

[B28] Janssen N. Barber H. A. (2012). Phrase frequency effects in language production. PLoS ONE 7:e33202 10.1371/journal.pone.0033202 22479370 PMC3314013

[B29] Jescheniak J. D. Schriefers H. Hantsch A. (2003). Utterance format affects phonological priming in the picture-word task: implications for models of phonological encoding in speech production. J. Exp. Psychol. Hum. Percept. Perform. 29, 441–454 10.1037/0096-1523.29.2.441 12760627

[B30] Konopka A. E. (2012). Planning ahead: How recent experience with structures and words changes the scope of linguistic planning. J. Mem. Lang. 66, 143–162 10.1016/j.jml.2011.08.003

[B30a] Kuipers J. R. La Heij W. (2009). The limitations of cascading in the speech production system. Lang. Cogn. Process. 24, 120–135 10.1080/01690960802234177

[B31] Laks B. (2005). La liaison et l'illusion in La Liaison de la Phonologie à la Cognition, eds Chevrot J. -P. Fayolle M. Laks B., *Langages* 158, 101–126 10.3917/lang.158.0101

[B32] Levelt W. J. M. (1989). Speaking: from Intention to Articulation. Cambridge, MA: MIT Press

[B33] Levelt W. J. M. Roelofs A. Meyer A. S. (1999). A theory of lexical access in speech production. Behav. Brain Sci. 22, 1–38 10.1017/S0140525X99001776 11301520

[B34] Martin R. C. Crowther J. E. Knight M. Tamborello F. P. Yang C. L. (2010). Planning in sentence production: evidence for the phrase as a default planning scope. Cognition 116, 177–192 10.1016/j.cognition.2010.04.010 20501338 PMC2930890

[B35] Meyer A. S. (1992). Investigation of phonological encoding through speech error analyses: achievements, limitations, and alternatives. Cognition 42, 181–211 10.1016/0010-0277(92)90043-H 1582156

[B36] Meyer A. S. (1996). Lexical access in phrase and sentence production: results from picture-word interference experiments. J. Mem. Lang. 35, 477–496 10.1006/jmla.1996.0026

[B37] Miozzo M. Caramazza A. (1999). The selection of determiners in noun phrase production. J. Exp. Psychol. Learn. Mem. Cogn. 25, 907–922 10.1037/0278-7393.25.4.907 10439500

[B38] Nespor M. Vogel I. (2007). Prosodic Phonology. Berlin: Mouton De Gruyter Originally published in 1986 (Dordrecht: Foris). 10.1515/9783110977790

[B39] Oppermann F. Jescheniak J. D. Schriefers H. (2010). Phonological advance planning in sentence production. J. Mem. Lang. 63, 526–540 10.1016/j.jml.2010.07.00416502144

[B39a] R Development Core Team (2005). R: A Language and Environment for Statistical Computing, Reference Index Version 2.x.x. Vienna: R Foundation for Statistical Computing ISBN: 3-900051-07-0. Available online at: http://www.R-project.org

[B40] Rossi M. Peter-Defare E. (1998). Les Lapsus ou Comment Notre Fourche a Langué. Paris: PUF

[B41] Schnur T. T. (2011). Phonological planning during sentence production: beyond the verb. Front. Psychol. 2:319 10.3389/fpsyg.2011.00319 22069396 PMC3208389

[B42] Schnur T. T. Costa A. Caramazza A. (2006). Planning at the phonological level during sentence production. J. Psycholinguist. Res. 35, 189–213 10.1007/s10936-005-9011-6 16502144

[B43] Schriefers H. Teruel E. (1999a). The production of noun phrases: a cross-linguistic comparison of French and German in Proceedings of the Twenty-First Annual Conference of the Cognitive Science Society, (Mahwah, NJ: Lawrence Erlbaum), 637–642

[B44] Schriefers H. Teruel E. (1999b). Phonological facilitation in the production of two-word utterances. Eur. J. Cogn. Psychol. 11, 17–50 10.1080/713752301

[B45] Stark E. Pomino N. (2009). Adnominal adjectives in romance. Where morphology seemingly meets semantics Proceedings of the IV Nereus International Workshop: Definiteness and DP Structure in Romance Languages, eds Espinal M. T. Leonetti M. McNally L. (Konstanz), 113–135

[B46] Thuilier J. Fox G. Crabbé B. (2012). Prédire la position des adjectifs épithètes en français. Lingvisticae Investigationes 35, 28–75 10.1075/li.35.1.02thu

[B47] Wagner V. Jescheniak J. D. Schriefers H. (2010). On the flexibility of grammatical advance planning during sentence production: effects of cognitive load on multiple lexical access. J. Exp. Psychol. Learn. Mem. Cogn. 36, 323–340 10.1037/a0018619 20192540

[B48] Waugh L. R. (1977). A semantic Analysis of Word Order: Position of the Adjective in French. Leiden: E. J. Brill

